# DNA Damage-Response Pathway Heterogeneity of Human Lung Cancer A549 and H1299 Cells Determines Sensitivity to 8-Chloro-Adenosine

**DOI:** 10.3390/ijms19061587

**Published:** 2018-05-28

**Authors:** Sheng-Yong Yang, Yi Li, Guo-Shun An, Ju-Hua Ni, Hong-Ti Jia, Shu-Yan Li

**Affiliations:** 1 Department of Biochemistry and Molecular Biology, Molecular Medicine and Cancer Research Center, Chongqing Medical University, Chongqing 400016, China; yangshengyong@cqmu.edu.cn (S.-Y.Y.); liyi@cqmu.edu.cn (Y.L.); 2 Department of Biochemistry and Molecular Biology, Beijing Key Laboratory of Protein Posttranslational Modifications and Cell Function, School of Basic Medical Science, Peking University Health Science Center, Beijing 100191, China; guoshunan@bjmu.edu.cn (G.-S.A.); juhuani@bjmu.edu.cn (J.-H.N.); jiahongti@bjmu.edu.cn (H.-T.J.)

**Keywords:** A549 cells, H1299 cells, heterogeneity, DNA damage response, 8-chloro-adenosine

## Abstract

Human lung cancer H1299 (p53-null) cells often display enhanced susceptibility to chemotherapeutics comparing to A549 (p53-wt) cells. However, little is known regarding to the association of DNA damage-response (DDR) pathway heterogeneity with drug sensitivity in these two cells. We investigated the DDR pathway differences between A549 and H1299 cells exposed to 8-chloro-adenosine (8-Cl-Ado), a potential anticancer drug that can induce DNA double-strand breaks (DSBs), and found that the hypersensitivity of H1299 cells to 8-Cl-Ado is associated with its DSB overaccumulation. The major causes of excessive DSBs in H1299 cells are as follows: First, defect of p53-p21 signal and phosphorylation of SMC1 increase S phase cells, where replication of DNA containing single-strand DNA break (SSB) produces more DSBs in H1299 cells. Second, p53 defect and no available induction of DNA repair protein p53R2 impair DNA repair activity in H1299 cells more severely than A549 cells. Third, cleavage of PARP-1 inhibits topoisomerase I and/or topoisomerase I-like activity of PARP-1, aggravates DNA DSBs and DNA repair mechanism impairment in H1299 cells. Together, DDR pathway heterogeneity of cancer cells is linked to cancer susceptibility to DNA damage-based chemotherapeutics, which may provide aid in design of chemotherapy strategy to improve treatment outcomes.

## 1. Introduction

The outcomes of DNA damage are diverse, depending on DNA damage types and DNA repair systems in the cell [1]. Among various types of DNA damage, double-strand breaks (DSBs) are the most lethal. DNA DSBs are induced by ultraviolet, ionizing radiation, and genotoxic chemicals or chemotherapeutics. DSBs can also occur upon replication of DNA containing single-strand breaks (SSBs) [1,2]. DNA damage triggers cell-cycle checkpoint and DNA repair mechanisms, which allow cells to repair damage. Defects in DNA repair result in genomic instability linked to increased risk of tumorigenesis [3]. On the other hand, DNA repair components of cancers can be targets for chemotherapy [4].

Central to the detection of DNA lesions is ATM (ataxia-telangiectasia mutated) and ATR (ATM- and Rad3-related) kinases, which get recruited to DNA damage sites and initiate DNA damage response (DDR). In the DSB-response cascade, ATM and ATR phosphorylate histone H2AX at Ser139 (γH2AX). γH2AX forms nuclear foci in the DNA domains next to the DSB over a megadalton distance and recruits DNA damage responsive proteins to integrate cell-cycle checkpoints and repair pathways in cells [1,2,3,4,5]. Meanwhile, ATM and ATR phosphorylate downstream kinases CHK2 and CHK1. ATM/CHK2- and ATR/CHK1-controlled checkpoints transiently arrest cells in G1, S or G2/M phases [2]. Arrest in G1, the dominant checkpoint response to DSBs, is mediated via p53. Activation of ATM/CHK2 and ATR/CHK1 leads to modification and stabilization of p53 protein [6]. The tumor suppressor p53 encoded by the TP53 gene transcribes downstream genes (e.g., p21^CIPI/WAF1^, p53R2, Gadd45, etc.), initiating DNA repair, growth arrest, senescence, and/or apoptosis [7,8,9].

Poly(ADP-ribose) polymerases (PARPs) are implicated in the regulation of chromatin structure, DNA replication, transcription and repair. PARP-1, a major member of PARP family, is activated by DNA strand breaks and involved in DNA repair such as homologous recombination (HR)-mediated SSB repair during replication and nonhomologous end joining (NHEJ)-mediated DSB repair in G1 [10]. Deficiency of PARP-1 leads to increased sensitivities of cells to DNA damage [11].

Cells with different single-gene depletion display distinctive sensitivity to chemotherapeutics [12]. Likewise, human non-small-cell lung cancer (NSCLC) H1299 (p53-null) cell is more sensitive to curcumin than A549 (p53-wt) cells [13]. In that work, the apoptotic molecules p53, bcl-2, and bcl-XL were examined, however, less is known about the link between DDR pathway heterogeneity and susceptibility to DNA damage in the two cancer cells. Our previous work has revealed that H1299 cells are more susceptible than A549 when exposed to 8-chloro-adenosine (8-Cl-Ado) [14], a potential anticancer drug currently in a phase I clinical trial for treatment of chronic lymphocytic leukemia. 8-Cl-Ado inhibits tumor cell proliferation and induces apoptosis by inhibiting DNA and RNA syntheses [15,16,17]. We have previously shown that 8-Cl-Ado induces DNA DSBs in human myelocytic leukemia K562 cell [18]. Since p53-dependent G1 arrest is critical to DNA repair, we are therefore concerned about the role of p53 in 8-Cl-Ado-induced DNA DSB response in p53-wt A549 and p53-null H1299 cells. We suppose that the heterogeneity of DNA DSB signal pathways in those cells might be linked to distinctive sensitivity to chemotherapeutics. To test this hypothesis, we comparatively investigated 8-Cl-Ado-induced DDR in A549 and H1299 cells and demonstrated that different accumulation of DNA DSBs and heterogeneity of DDR pathways determine their distinctive susceptibilities to 8-Cl-Ado, which may provide aid in the design of future chemotherapy strategies to improve treatment outcomes.

## 2. Results

### 2.1. H1299 Cells Are More Sensitive to 8-Cl-Ado-Induced Growth Inhibition and Apoptosis than A549 Cells

**Figure 1:**
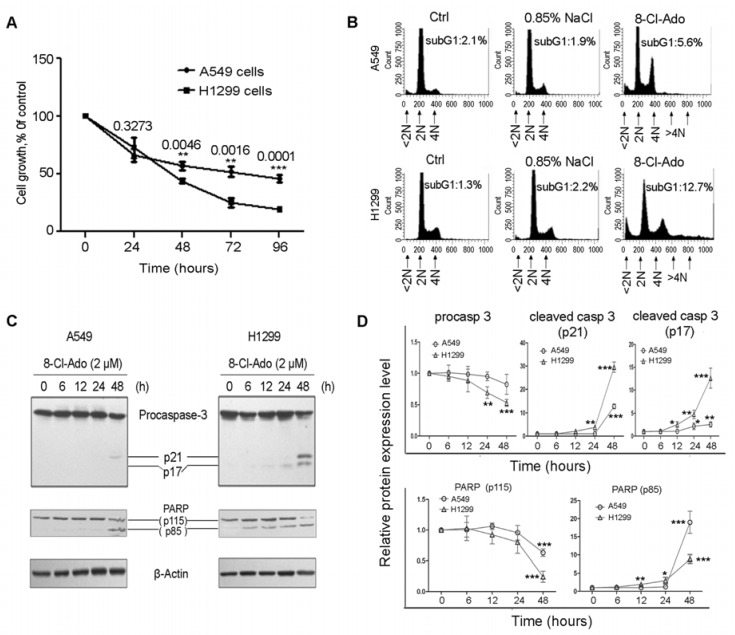
Effects of 8-Cl-Ado on cell growth and apoptosis. A549 and H1299 cells were exposed to 2 μM 8-Cl-Ado for indicated hours. (**A**) Cell proliferation was evaluated with MTT assay (see materials and methods). Data represent mean ± SD (*n* = 3); (**B**) cells exposed to 8-Cl-Ado for 48 h were stained with propidium iodide whose signal was measured by FACScan. Apoptotic cells (subG1/<2N) were assayed by the computer program CELLQuest. Data are representative of three independent experiments; (**C**) a representative Western blotting for Procaspase-3 activation and PARP-1 cleavage in 8-Cl-Ado-exposed cells. β-Actin as a loading control; (**D**) relative levels of Procaspase-3, Procaspase-3-cleaved fragments (p21 and p17), PARP-1 (p115) and its cleaved product (p85) in Western blotting. The blots were screened/quantified with the software Quantity One (Bio Rad) and normalized against β-Actin level, and the ratio of target protein to Actin from control (0 h exposure) cells was designated as “1” (100%). Data represent mean ± SD (*n* = 3). * *p* < 0.05; ** *p* < 0.01; *** *p* < 0.001.

As shown in Figure 1A, 8-Cl-Ado (2 μM) significantly inhibited both A549 (p53-wt) and H1299 (p53-null) cell proliferation after 48 h exposure; the inhibitory rates in H1299 cells at 48, 72 and 96 h were 57%, 75% and 81%, respectively, which were much higher than 44%, 48% and 51% inhibitory rates in A549 at the same time points. Flow cytometry showed that more apoptotic cells (subG1/<2N) occurred in H1299 (12.7%) than A549 (5.6%) after 48 h 8-Cl-Ado exposure (Figure 1B). Consistently, the active caspase-3 subunits p17 and p21 from procaspase-3, a sign of apoptosis, and the p85 fragment produced by the cleavage of caspase-3 substrate PARP-1 (115 kD) were detectable in H1299 cells after 12–24 h exposure, but a weak activation of procaspase-3 and cleavage of PARP-1 were not seen in A549 cells until 48 h after exposure (Figure 1C,D). These results suggest that H1299 cell is more sensitive to 8-Cl-Ado-induced growth inhibition and apoptosis than A549.

### 2.2. 8-Cl-Ado Diminishes PARP-1-Associated TOPO I Activity and p53R2 Expression in H1299 Cells More Greatly than A549 Cells

**Figure 2:**
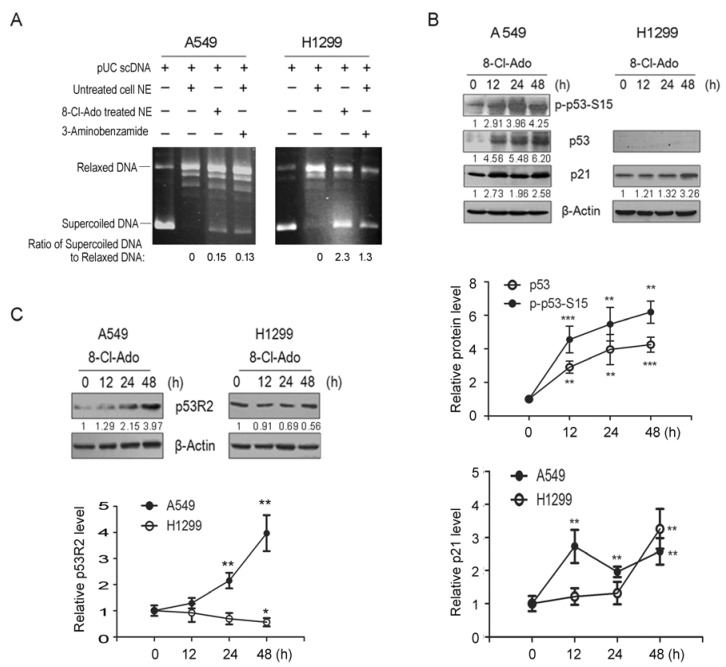
Effects of 8-Cl-Ado on DNA relaxation and on p53, p21 and p53R2 expression. (**A**) A549 and H1299 cells were exposed to 2 μM 8-Cl-Ado for 48 h, and nuclear extracts (NE) were prepared. Relaxation activities in NE were tested by incubating with supercoiled pUC19 DNA in the reaction conditions as indicated on the top. After ethanol precipitated, extracted DNA samples were subjected to 1% agarose gel electrophoresis. The pUC19 DNA is used as markers for supercoiled and relaxed DNA; (**B**,**C**) Western blotting for p53, p21 and p53R2 expression. β-Actin as a loading control. The numbers below the blots and histograms in lower panels show the relative levels of p53, p21 and p53R2 in Western blotting. The ratio of target protein/Actin from control cells was designated as “1”. * *p* < 0.05; ** *p* < 0.01; *** *p* < 0.001.

Since PARP-1 can stimulate topoisomerase I (TOPO I)-like activity [11,19] that can relax negatively supercoiled DNA and convert it to a relaxed form, we performed DNA relaxation assays to examine the effect of PARP-1 cleavage on TOPO I-like activities in A549 and H1299 cells. In these assays, supercoiled pUC19 plasmid DNA was used as substrate and incubated with nuclear extracts (NE) from 8-Cl-Ado-treated or untreated cells. In the reactions containing NE from untreated A549 and H1299 cells, the ratio of supercoiled DNA to relaxed DNA approximates to zero (Figure 2A, lane 2), indicating that nearly all supercoiled DNA was transformed into relaxed DNA and high constitutive activities of TOPO I was present in the 8-Cl-Ado-untreated nuclei. Inhibition of TOPO I activities in the NE from 8-Cl-Ado-treated A549 and H1299 cells was evidenced by the partially remnant supercoiled DNA. Notably, the remnant of supercoiled DNA (2.30, the ratio of supercoiled DNA to relaxed DNA) in exposed-H1299 NE was much more than that (0.15) in exposed-A549 NE (lane 3); in other words, the inhibitory TOPO I activity in exposed H1299 cells was 15-fold of exposed A549 cells. The inhibition of TOPO I-like activities in exposed cells was attributed at least in part to suppressing PARP-1, because inhibitory TOPO I was detectable when added the specific PARP inhibitor 3-aminobenzamide (3-AB) to unexposed NE (Figure 2A, lane 4). These results support the notion that PARP-1 is functionally associated with TOPO I activity [19,20]. These data also indicate that based on the disruption of PARP-1 by caspase-3 (Figure 1C), TOPO I-like activity in p53-null H1299 cells is lost much more than p53-wt A549 cells during 8-Cl-Ado exposure.

Next, we tested expression of p53/TP53 and its targets p21 and p53R2 in both cells. As expected, following S15-phosphorylation of TP53 and its accumulation (Figure 2B, upper and middle panels), the level of TP53-dependent p21 protein was greatly increased (Figure 2B upper and lower panels) in A549 within 12–48 h after 8-Cl-Ado exposure. In H1299 cells, however, TP53-independent p21 was significantly increased only after 48 h exposure (Figure 2B, upper and lower panels), because H1299 is TP53-null. The levels of p53R2 were greatly stimulated in A549 but a constitutive p53R2 was downregulated in H1299 upon drug exposure (Figure 2C). These results indicate that p53-dependent p21 and p53R2 expression in A549 cells are more active than that in H1299 cells under 8-Cl-Ado exposure.

### 2.3. 8-Cl-Ado Induces More Accumulation of DSBs in H1299 Cells than in A549 Cells

**Figure 3:**
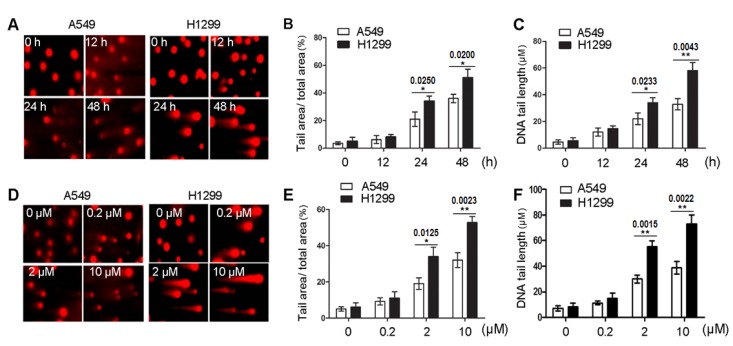
Comet assays for 8-Cl-Ado-induced double-strand breaks (DSBs). (**A**) A typical representation of time-dependent comet assays. A549 and H1299 cells were exposed to 2 μM 8-Cl-Ado for indicated hours. DSBs were evaluated by the percentage of DNA tail area in whole DNA area (**B**) and by comet tail length (**C**); (**D**) a representative dose-dependent comet assays. Cells were exposed to increased 8-Cl-Ado for 48 h; (**E**) the percentage of DNA tail area in whole DNA area; and (**F**) the comet tail length. Data represent mean ± SD (*n* = 3). The percentage of comet tail area and tail length was analyzed in at least 50 cells each slide. * *p* < 0.05; ** *p* < 0.01.

**Figure 4:**
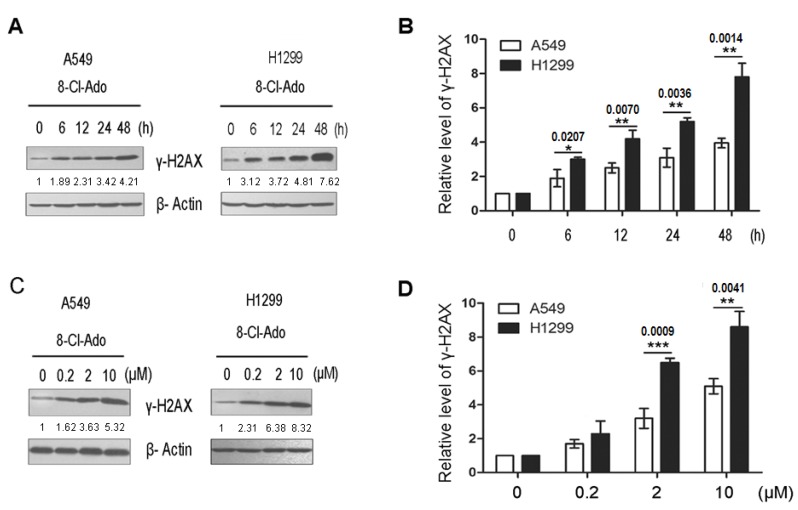
Western blotting and immunocytochemistry for γ-H2AX expression in A549 and H1299 cells. (**A**) Cells were exposed to 2 µM 8-Cl-Ado for 0, 6, 12, 24 and 48 h. Western blotting was performed with specific antibody. β-Actin as a loading control; (**B**) histograms showing the relative levels of γ-H2AX in (**A**) experiments. The ratio of γ-H2AX to Actin at 0 h is normalized to “1”. Data represent mean ± SD (*n* = 3); (**C**) cells were exposed to 8-Cl-Ado at the indicated concentrations for 48 h, and Western blotting was performed for a dose-dependent increase of γ-H2AX; (**D**) histograms showing the relative levels of γ-H2AX in (**C**) experiments. * *p* < 0.05; ** *p* < 0.01; *** *p* < 0.001.

DNA DSBs can arise from replication of DNA containing single-strand breaks (SSBs) [1,2]. Moreover, inhibition of TOPO I activity induces SSBs [20], and p53R2 participates urgent DNA repair [8]. To investigate if loss of TOPO I-like activity and downregulation of p53R2 may promote 8-Cl-Ado-induced DSBs in H1299, comet assays were employed to test accumulation of DSBs in both cells under the condition of exposure to 2 µM 8-Cl-Ado for 0, 12, 24 and 48 h (Figure 3A–C) or to increased concentration of 8-Cl-Ado (0, 0.2, 2 and 10 µM) for 48 h (Figure 3D–F). The percentage of DNA tail area (“TA”) to DNA whole area (“WA”) (Figure 3B,E), and DNA tail length (Figure 3C,F) in both exposed cells were increased in a time- (Figure 3B,C) and dose-dependent (Figure 3E,F) manner. In the time-dependent effects, the percentages of “TA” to “WA” at 24 and 48 h were 20.9% and 36.6% in A549 cells, whereas 34.8% and 52.2% in H1299 cells, respectively (Figure 3B). In the dose-dependent effects, the percentages of “TA” to “WA” under 2 µM and 10 µM 8-Cl-Ado exposure were 19.4% and 31.8% in A549 cells, while 34.4% and 52.9% in H1299 cells (Figure 3E). The average tail lengths were 30 ± 3 μm in A549 cells and 55 ± 5 μm in H1299 cells when exposed to 2 µM 8-Cl-Ado for 48 h (Figure 3C), and 39 ± 4 μm in A549 cells but 73 ± 6 μm in H1299 cells when exposed to 10 µM 8-Cl-Ado for 48 h (Figure 3F). More accumulation of DSBs in H1299 cells than in A549 cells was also quantitatively evaluated by Western blotting for γ-H2AX expression (Figure 4A–D). These results indicate that 8-Cl-Ado induced more DSB accumulation in H1299 cells than A549, which should be related at least partly to the inhibition of TOPO I-like activity and p53R2 expression.

### 2.4. Defect in p53-p21 Signal in H1299 Cells Leads to Increased S Subpopulation upon DSBs

**Figure 5:**
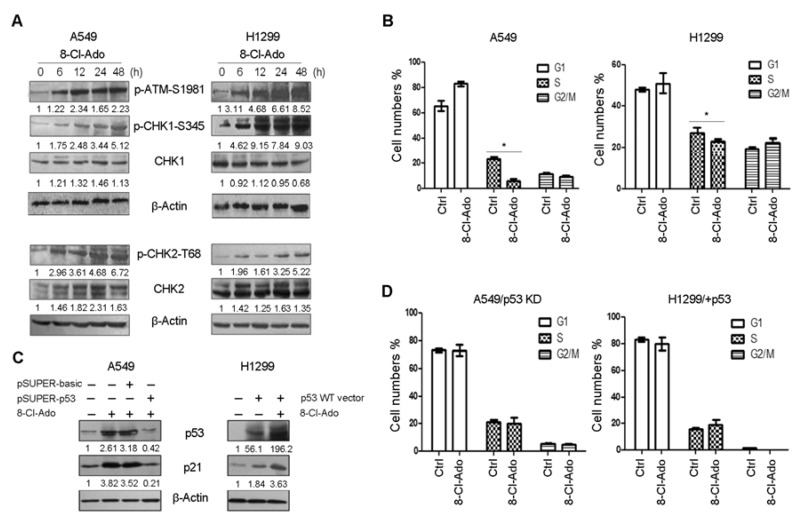
Signal pathways and cell-cycle progression in DDR (DNA damage response). (**A**) A549 and H1299 cells were exposed to 2 μM 8-Cl-Ado for indicated hours, and Western blotting was performed for components of signal-transduction pathways. The relative levels of target proteins were normalized against β-Actin; (**B**) cell-cycle analysis. Cells were exposed to 2 μM 8-Cl-Ado for 48 h. After harvested and fixed, cells were stained with propidium iodide (PI); PI signal was measured by FACScan. G1, G2/M and S populations in the cell-cycle were analyzed by computer programs. Data present ± SD (*n* = 3). * *p* < 0.05; (**C**) Western blotting for p53 and p21 in p53-silenced A549 and p53-overexpressed H1299 cells. Cells were transiently transfected with pSUPER-basic (control), pSUPER-p53 (for silencing TP53), or p53-WT expression plasmid for 48 h and exposed to 8-Cl-Ado for additional 48 h, followed by Western blotting. The relative levels of target proteins were normalized against β-Actin; (**D**) G1 and G2/M and S subpopulations in p53-silenced A549 cells and p53-overexpressed H1299 cells.

We first examined cell cycle checkpoint signals in DDR. Phospho-ATM-S1981 was significantly increased within 48 h upon 8-Cl-Ado exposure in both cells (Figure 5A). Following ATM activation, phospho-CHK1-S345 and phospho-CHK2-T68 were increased within 6–48 h in both H1299 and A549 cells. It seemed that CHK1 phosphorylation dominantly occupied in H1299 cells, whereas CHK2 phosphorylation predominated in A549 cells. This difference between the two cells presumably is attributable to more SSBs induced by TOPO I inhibition in H1299 cells.

After 48 h exposure, A549 cells increased G1 subpopulation from 65 (control) to 84% but decreased S phase cells sharply from 23 to 7% and had no significant changes in G2/M cells (from 10 to 9%), while H1299 cells increased G1 phase cells from 47 to 52%, decreased S phase cells from 28 to 23%, and still remained 22% G2/M cells (Figure 5B). Alternatively, the percentages of S phase and G2/M phase cells in 48 h-exposed H1299 cells are threefold and twofold as much as in exposed A549, respectively, indicating that even in the presence of DSBs, H1299 cells had more cells entering into S and G2/M phases. Not surprisingly, induction of p21 followed after p53 phosphorylation/accumulation in exposed A549 cells, but no marked increase until 48 h in exposed H1299 cells (Figure 2B). Following silence of p53 by RNA interference (RNAi) in A549 cells (Figure 5C, left panel) or over-expressed p53 in H1299 cells (Figure 5C, right panel), p21 was down- or upregulated (Figure 5C). Similar G1, S and G2/M subpopulations occurred in p53-silenced A549 (Figure 5D, left panel) and p53-overexpressed H1299 (Figure 5D, right panel) under exposed and unexposed conditions; it was much the same of G1, S and G2/M subpopulations in unexposed A549 and p53-overexpressed H1299 cells, but a little difference between them under exposed condition, probably due to over-expressing of exogenous p53. At any rate, these results indicate that p21 increases G1 phase but restricts S and G2/M phase cells. Together, defect in p53-p21 signal leads to more serious impairment of G1 checkpoint and to more S phase cell accumulation in H1299 cells than in A549 cells during DDR.

### 2.5. 8-Cl-Ado-Induced More Accumulation of DSBs in H1299 Is Associated with DNA Replication in S Phase

**Figure 6:**
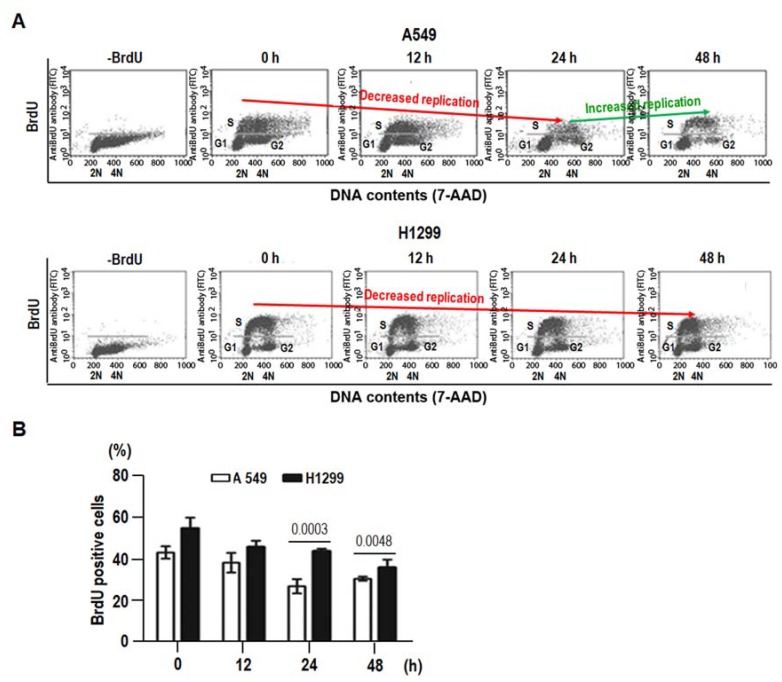
BrdU incorporation into 8-Cl-Ado-exposed A549 and H1299. After exposed to 2 μM 8-Cl-Ado for indicated hours, cells were pulsed with BrdU prior to harvest. After DNase treatment, cells were stained with fluorescein isothiocyanate (FITC)-conjugated anti-BrdU antibody, followed by flow cytometry with FACS Diva software (BD Biosciences, San Jose, CA, USA). (**A**) A representative flow cytometry analysis of BrdU-positive cells. S, G1 (2N) and G2/M (4N) cells are indicated; (**B**) histograms showing the percentage of BrdU-positive S cells in flow cytometry experiments. Data represent mean ± SD (*n* = 3).

DNA DSBs interfere with DNA replication [1]. We thus compared DNA synthesis in both cells using BrdU incorporation. In consistence with the results shown in Figure 5B, more BrdU-labeled S and G2 cells in H1299 cells than A549 cells were detectable after 24 h 8-Cl-Ado-exposure (Figure 6). DNA synthesis was continually decreased in H1299 cells within 12–48 h of exposure, but only seen at earlier steps (<24 h) in A549 cells (Figure 6A). The percentages of BrdU-incorporated S cells in A549 cells after 0, 12, 24 and 48 h exposure were 44.6%, 38.2%, 28.7% and 32.5%; in other words, DNA synthesis was continually decreased before 24 h but became increased by 48 h, indicating that DNA repair capability initiates a little recovery within 24–48 h. In H1299, however, the percentages of BrdU positive S cells at the same time-points were 54.9%, 48.2%, 46.7% and 38.7%, respectively. Importantly, the BrdU-incorporated rates at 24 and 48 h in H1299 were significantly higher thanA549 (Figure 6B). The continual drops of BrdU-incorporated S cells in H1299 cells suggest that DNA damage remains at all times and the repair capability is unrecovered, probably due to p53 defect and p53R2 reduction. In addition, more DNA synthesis in S phase may result in more DSBs in H1299 cells.

### 2.6. DNA Damage Response Proteins Are Time-Differentially Mobilized in H1299 and A549 Cells during DSBs

**Figure 7:**
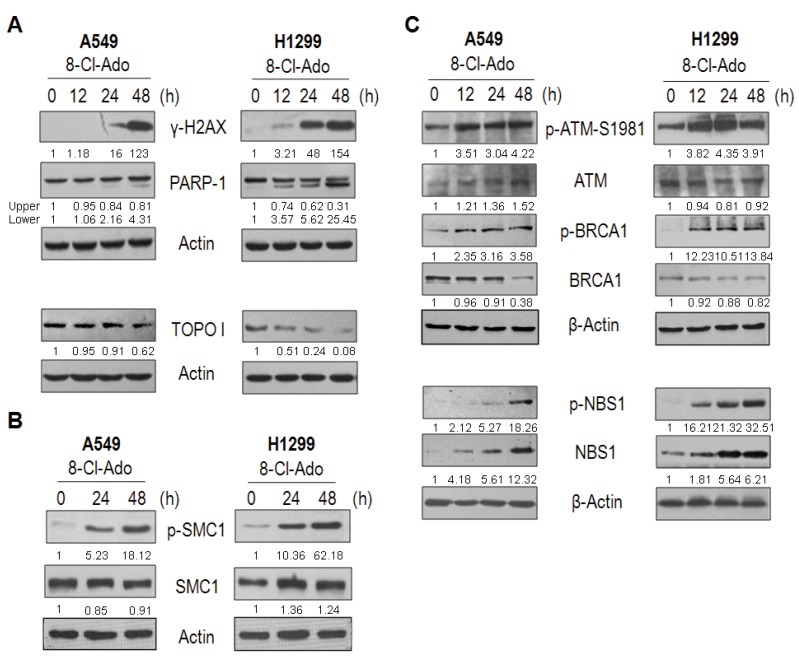
Western blotting for PARP-1, TOPO I and DNA responsive proteins in DDR. A549 and H1299 were exposed to 2 μM 8-Cl-Ado for the indicated hours. The γ-H2AX, PARP-1 and TOPO I (**A**), SMC1 (**B**), ATM, BRCA1 and NBS1 and their modifications (**C**) were analyzed by Western blotting with specific antibodies. Also see Figure S1 for BRCA1/pBRCA1 full blots of A549 cells in (**C**). β-Actin as a loading control. The ratio of target protein/Actin from control cells was designated as “1”.

To check the difference between DNA repair pathways in H1299 and A549 cells, Western blot was performed to determine the expression of DNA repair factors and enzymes. As shown in Figure 7, the expression and phosphorylation/activation of ATM, BRCA1 and SMC1 in DDR presented similar dynamics in both cells (Figure 7B,C), while the expression dynamics of PARP-1, TOPO I, NBS1/phospho-NBS1 displayed differently in both cells. Obvious cleavage of PARP-1 was observed again in H1299 rather than A549 cells within 12–48 h after 8-Cl-Ado exposure (Figure 7A, also see Figure 1C,D). Following PARP-1 cleavage, TOPO I was greatly downregulated at the same time-points in H1299 cells, but only a little drop by 48 h in A549 cells. The phosphorylation of NBS1 at Ser343 in H1299 was earlier and stronger than A549 (Figure 7C).

## 3. Discussion

Computational biology study reveals that intratumor signaling heterogeneity or pathway dysregulation is associated to clinical outcome of cancers [21]. The DDR signal pathway is composed of a cell cycle checkpoint and DNA repair mechanisms that maintain genomic stability in normal cells and can also serve as targets for cancer chemotherapy. Our hypothesis that the signaling pathway heterogeneity of cellular response to DSBs might determine chemotherapeutic sensitivity of cancer cells is evidenced in this study, and our major finding is that cancer cells (e.g., H1299) lacking ATM-CHK2-p53-p21 mediated G1 checkpoint and p53-dependent DNA repair are much more sensitive to chemotherapeutics. In other words, p53-p21 signal may at least in part protect p53-wt cancer cells (e.g., A549) from DNA damage stress. Our finding support the notion that p53 signaling suppresses apoptosis following genotoxic stress, facilitating repair of genomic injury under physiological conditions but having the potential to promote tumor regrowth in response to cancer chemotherapy [22].

Initial studies of cellular response to anticancer drugs suggested that p53-dependent apoptosis was the common mechanism of cancer chemotherapy. Subsequent work on p53-null cells and animal models, however, argued that genotoxic agents could also induce significant cytotoxicity in a p53-independent manner [23,24,25]. Indeed, p53-null H1299 cells were more sensitive to p53-independent apoptosis than p53-wt A549 cells when exposed to curcumin [13], which inhibits cell cycle and cell survival by inducing DNA damage [26]. Similarly, we found that H1299 cells were more sensitive to 8-Cl-Ado-inducd growth inhibition and apoptosis than A549 cells [14] (also see Figure 1A,B). It seemed that hypersemsitivity of H1299 was linked to 8-Cl-Ado induced DSBs, because 8-Cl-Ado induced more severe DSBs in H1299 than A549 (Figure 3 and Figure 4). Several reasons may account for more extensive and severe DSBs in H1299 than A549 cells.

First, p53-p21 signal deficiency and S cell accumulation by SMC1 activation presumably contributes to more DSBs in H1299 cells than A549 cells. After detection of DSBs by ATM, p53 is phosphorylated/activated and arrests cells in G1 via activating p21 gene expression [6,7,8]. p53-induced p21 not only induces G1 arrest but inhibits DNA replication without interfering with DNA repair through binding to the replication/repair factor PCNA [27] and PARP-1 [28] in DDR. We found that in A549 cells, the p21 protein was rapidly up-regulated following p53 activation and strictly arrested most cells in G1 phase upon DSBs, while p53-null H1299 cells had a delayed induction of p21 only by 48 h, leading to G1 checkpoint loss and more S cell accumulation (Figure 5). Also, more S cell accumulation in H1299 might be attributed to SMC1 phosphorylation, because phosphorylation of SMC1 at Ser957 is required for intra-S checkpoint [29,30]. During DDR, SMC1 is phosphorylated by ATM/ATR in the presence of BRCA1 and NBS1 [31]. Activating intra-S checkpoint and inhibiting TOPO I can increase DSBs [20,22], which arise from replication of DNA containing SSBs [1,2]. We did find stronger inhibition of TOPO I (Figure 2A and Figure 7A) and activation of SMC1 followed by BRCA1 and NBS1 activation at 24 h after 8-Cl-Ado exposure in H1299 cells (Figure 7C), and more accumulation of S (BrdU positive) cells in H1299 (Figure 5B and Figure 6). The S cells with uncovered capability of DNA synthesis are particularly vulnerable to DNA damage, which may cause replication stress, then replication-stress-induced DSBs. Previous notion [29,30,31] and our data can explain why more DSBs occur in H1299 than A549.

Second, defects of p53 and p53-dependent DNA repair capability are associated with more DNA DSBs and apoptosis in H1299 than A549. DNA DSB is repaired by NHEJ in G1 phase and HR in late S and G2 [1,2,3,4,5]. The p53 protein guards genomic stability through direct or indirect roles in DNA repair. For instance, p53 modulates Holliday Junctions and broken end reconnecting and annealing in HR repair [4]. The protein can also interact with repair proteins such as replication protein A (RPA), Rad51 and Rad52 to promote HR repair [32]. In addition to p21, p53 as a transcription factor can also promote controlling DNA repair gene expression, such as BRCA1, p53R2, GADD45 and PCNA. For instance, p53 can transcriptionally activate BRCA1 expression [7], and in turn, ATM-phosphorylated BRCA1 interacts with and enhances p53 transactivation function [6,7,32]. BRCA1 selectively co-activates p53-dependent genes such as p21 and p53R2 [8,33] targeting DNA repair and cell cycle arrest but not apoptosis [34]. This is because that p21-PCNA interaction inhibits DNA replication [27]; p21 association with PARP-1 blocks replication fork progression [28]; p53-dependent p21 can also bind procaspase-3 to protect cells from apoptosis [34]. Moreover, p53-induced p53R2 supplies dNTPs for urgent DNA repair during G1 and G2 arrests [8,33]. In our case, p53R2 as a direct target for p53 was strongly induced in A549 cells (Figure 2C), which might promote the capability of DNA repair and could be associated with a lesser increase of DSBs in A549 cells than H1299 cells. In addition, transcription factor E2F1 promotes G1/S transition and induces apoptosis by controlling target gene expression [35]. We have previously shown that E2F1 is induced in H1299 cells during DDR [36,37]. E2F1 may therefore counteract p21-inhibited G1/S transition, promoting S phase cells and apoptosis in H1299 cells. However, E2F1 cannot achieve that in A549 cells, because p53 may counteract E2F1 effect by association with it [38]. Contrarily, the early nucleolar accumulation of E2F1 may release p53 function [36] to activate p21 and p53R2 genes and p53-dependent DNA repair in A549 cells. Indeed, A549 cells displayed a partial capability of recovering DNA replication at late time of 8-Cl-Ado exposure, but H1299 cells could not (Figure 6). All above-mentioned can explain more G1 cells and less DNA DSBs in A549 cells, but more S phase cells and DSBs in H1299 cells.

Third, loss of PARP-1 activity by caspase-3 cleavage is linked to more accumulation of DNA DSBs in H1299 than A549. PARP-1 is activated by DNA strand breaks and functions as a positive regulator in DNA repair. PARP-1 mediates DSB end-joining in mammalian cells, which may complement the DNA-PK/XRCC4/ligase IV-dependent NHJE [39]. PARP-1-mediated DSB end-joining depends on its interaction with repair proteins, by which PARP-1 recruits repair proteins/enzymes to DSB sites [10]. Contrarily, the small cleaved fragment (24-kDa) from PARP-1 blocks the access of repair proteins/enzymes to DSBs [40], indicating that PARP-1 cleavage impairs its capability of recruiting repair proteins/enzymes. Moreover, PARP-1 can stimulate the activity of DNA-PK for NHJE in G1 [10]. In addition, PARP1 is required for rapid recruitment of MRE11 and NBS1 at DSB sites during HR repair in S phase [41]. Therefore, deficiency of PARP-1 may increase sensitivities of cells to DNA damage agents [10]. Also, PARP-1 can promote TOPO I and TOPO I-like activity [11,19] and reactivate stalled TOPO I activity [42], therefore loss of PARP1 may decrease DNA repair capability and increases SSBs and SSB-containing DNA replication-mediated DSBs. We thus conclude that loss of PARP-1 by caspase-3 cleavage is accounted for more DSBs in H1299 than A549. Our data suggest that inhibition of PARP-1 as well as TOPO I may sensitize cancer cells to chemotherapeutic agents in certain situations.

p53 (TP53) acts as a tumor suppressor by orchestrating various signaling pathways, in which the activity of p53 involves several positive and negative feedback loops that determine the cell fates through cell cycle arrest, DNA repair or apoptosis [43]. Growing evidence suggests that p53 can act as a tumor suppressor via p53-microRNA loops. Genome-wide screen for microRNAs revealed that many TP53 targeted miRNAs including miR-34a have been implicated in p53-mediated apoptosis during DDR [44]. Most recently, a study showed a positive p53/Wip1/miR-16 feedback loop for G1/S checkpoint during DNA damage [45]. Therefore, we cannot exclude the participation of p53 and microRNA feedback loops in 8-Cl-Ado-induced DSB response in A549, which might contribute to differential sensitivities of A549 and H1299 cells to the drug.

In summary, we tested our hypothesis that more extensive and severe DNA damage was linked to higher sensitivity of H1299 to 8-Cl-Ado treatment, whereas less DNA damage was linked to lower sensitivity of A549. We have clarified the major causes of more extensive DSBs in H1299 cells. Together, the heterogeneity of DDR signaling pathways determines the sensitivity of cancer cells to DNA damage-based chemotherapeutics. Notably, we comparatively investigated the effects of 8-Cl-Ado on NSCLC H1299 and A549 cells, whether our finding is suited to other genotoxic agents and cancer cells remains to be clarified. In addition, we examined only some of the key molecular components of the DDR signaling pathways; gene chip analysis is needed for detailed knowledge of the condition in the future.

## 4. Materials and Methods

### 4.1. Cell Culture and Treatment

Human lung cancer A549 (p53-wt) and H1299 (p53-null) cells from ATCC (Manassas, VA, USA) were cultured in Dulbecco minimum essential medium (DMEM, Gibco, Grand Island, NY, USA) supplemented with 10% fetal bovine serum (Gibco, Grand Island, NY, USA), 100 U/mL penicillin and 100 mg/mL streptomycin, and grown at 37 °C with 5% CO_2_. 8-Chloro-adenosine (8-Cl-Ado) (the State Key Laboratory for Natural and Biomimetic Drugs, Peking University HSC, Beijing, China) was dissolved in 0.9% NaCl solution in given concentrations.

### 4.2. Cell Proliferation Assay

Cells were cultured in 96-well plates (15,000 cells/0.2 mL per well). 8-Cl-Ado (2 μM) was added to cultures, followed by incubation for given hours. Before harvest, 20 μL MTT (3-(4,5-dimethythiazolzyl)-2,5-diphenyl tetrazolium tromide, 5 mg/mL; Sigma, St. Louis, MO, USA) was added to each well. After incubating for 4 h, 0.2 mL dimethyl sulfoxide (DMSO) was added to terminate reactions. Absorbance values were determined spectrophotometrically at 490 nm on a Microplate Reader (BIO-TEK, Rockville, MA, USA).

### 4.3. Flow Cytometry Analysis

Typically, 1 × 10^6^ cells were collected, washed twice in ice-cold PBS and fixed in ice-cold 70% ethanol overnight at 4 °C. Then cells were washed twice in ice-cold PBS and digested with RNase A (10 µg/mL) at 37 °C for 30 min. Cells were stained with 10 µg/mL of propidium iodide (Sigma) for 3 min at room temperature before testing. DNA contents of cells (10,000 cells per experimental group) were analyzed using computer programs CELLQuest and ModFit LT 2.0ep for Power (Becton Dickinson, Franklin Lakes, NJ, USA). Apoptosis was assayed by the appearance of a sub-G1 (<2N ploidy) population by the computer program CELLQuest (Becton Dickinson, Franklin Lakes, NJ, USA).

### 4.4. DNA Relaxation

Reaction mixtures containing 0.4 mg pUC19 plasmid DNA (MBI Fermentas, Vilnius, Lithuania) and 2.5 μg nuclear extracts (NE) from 8-Cl-Ado-exposed or -unexposed cells, or 5 mM 3-aminobenzamide (PARP inhibitor) in 20 μL relaxation buffer (50 mM Tris-HCl, pH 8.0, 0.1 M NaCl, 5 mM MgCl_2_) were incubated at 37 °C for 30 min and stopped by adding sodium dodecyl sulphate (SDS) and ethylenediaminetetraacetic acid (EDTA) to a final concentration of 0.1% and 10 mM, respectively. DNA was ethanol precipitated, and subjected to electrophoresis in 1% agarose gels. DNA was stained with 1 mg/mL ethidium bromide and visualized by ultraviolet (UV) light.

### 4.5. Comet Assay

As described previously [46], a 80 μL mixture containing 10^5^ cells treated with or without 8-Cl-Ado in 40 μL PBS, and 40 μL 1% low melting point agarose (final concentration 0.5%) was pipetted onto the first agarose layer of the full-frosted microscope slides that were precoated with 0.5% normal melting point agarose. After lysis for 2 h at 4 °C in fresh lysing solution, slides were placed in a horizontal gel electrophoresis unit filled with fresh electrophoresis solution for 20 min. Following unwinding, electrophoresis was performed for 20 min at 0.7 V/cm (300 mA/25 V) at 4 °C. After electrophoresis, slides were neutralized twice with 0.4 M Tris buffer (pH 7.5) for 15 min. Slides were stained with ethidium bromide for 10 min in the dark. After staining, slides were examined at 600× magnification, and pictures were taken under fluorescence microscope (Leica, Mannheim, Germany). To score the percentage of DNA in the tail, the image analysis system was used (Q550CW; Leica, Wetzlar, Germany). The percentage of comet tail area (the ratio of DNA tail area to total DNA area) and comet tail length (from the center of the DNA head to the end of the DNA tail) was analyzed in 50 cells for one slide.

### 4.6. Constructs and Transfection

pSUPER-p53 plasmid was constructed by ligating the annealed primers 5′-GATCCCCGACTCCAGTGGTAATCTACTTCAAGAGAGTAGATTACCACTGGAGTCTTTTTA-3′, 5′-AGCTTAAAAAGACTCCAGTGGTAATCTACTCTCTTGAAGTAGATTACCACTGGAGTCGGG-3′ into the *Bgl* II and *Hind* III sites of pSUPER-basic (OligoEngine, Seattle, WA, USA), and correct plasmid was confirmed by direct sequencing. p53 wild-type plasmid was a gift from Dr. Bert Vogelstein (Johns Hopkins University, Baltimore, MD, USA). Transfection was performed with Lipofectamine 2000 (Invitrogen, Carlsbad, CA, USA) following manufacturer’s protocol.

### 4.7. Western Blotting

Whole-cell extracts were prepared in lysis buffer and protein concentration was determined using the BCA Protein Assay Reagent Kit (Pierce, Rockford, IL, USA). Fifty micrograms of total proteins were loaded onto 10–13% sodium dodecyl sulphate-polyacrylamide gel electrophoresis (SDS-PAGE), and transferred onto nitrocellulose membranes. Membranes were incubated with primary antibodies overnight at 4 °C with gentle rocking followed by horseradish peroxidase-conjugated secondary antibody for 1 h. Chemiluminescence signals were visualized using Western blotting luminol reagent (Santa Cruz Biotech, Santa Cruz, CA, USA) and exposed to film. The blots were screened/quantified with the software Quantity One (Bio Rad, Hercules, CA, USA) and normalized against β-Actin level. The target protein/Actin value obtained from control (8-Cl-Ado-exposed for 0 h) cells was designated as “1”. Anti-p21, anti-p53, anti-p53R2, anti-phospho-p53-S15, anti-CHK1-S345, anti-CHK2-T68, anti-CHK1, anti-CHK2, anti-ATR, anti-phospho-ATR-S428, anti-NBS1, anti-phospho-NBS1-S343, anti-SMC1, anti-phospho-SMC1-S699, anti-β-actin, anti-BRCA1 and anti-phospho-BRCA1-S1524 antibodies were purchased from Cell Signaling Technology; anti-phospho-histone H2AX-S139, anti-ATM and antiphospho-ATM-S1981 were acquired from R&D Systems Inc. (Minneapolis, MN, USA).

### 4.8. BrdU Incorporation Assay

As previously described [18], BrdU incorporation was performed using the fluorescein isothiocyanate (FITC) BrdU Flow Kit (BD Pharmingen, San Diego, CA, USA), according to the manufacturer’s instructions. After exposed to 2 μM 8-Cl-Ado, cells were pulsed with final concentration of 10 μM BrdU for 30 min at 37 °C prior to harvest. Cells were washed in cold staining buffer (1 × Dulbecco’s phosphate-buffered saline +3% FBS), fixed/permeabilized with Cytofix/Cytoperm buffer and washed with Perm/Wash buffer (on ice). Cells were treated with 30 μg DNase for 1 h at 37 °C, and stained with FITC-conjugated anti-BrdU antibody and 7-AAD. DNA contents were analyzed by a FACSCanto flow cytometer with FACSDiva software (BD Biosciences, San Jose, CA, USA).

### 4.9. Statistical Analysis

The Student’s *t*-test and ANOVA test were used for univariate analysis. Statistical significance was defined by a two-tailed *p*-value of 0.05.

## Supplementary Materials

Supplementary materials can be found at http://www.mdpi.com/1422-0067/19/6/1587/s1.
