# Supplementary material for: DNA Damage-Response Pathway Heterogeneity of Human Lung Cancer A549 and H1299 Cells Determines Sensitivity to 8-Chloro-Adenosine
Source: Int J Mol Sci. 2018 May 28;19(6):1587. doi: 10.3390/ijms19061587 (PMC6032248; doi:10.3390/ijms19061587)
Supplement: Supplementary file 1 [file ijms-19-01587-s001.pdf]

Int. J. Mol. Sci. **2018**, *19*, 1587; doi:10.3390/ijms19061587

Supplementary Materials

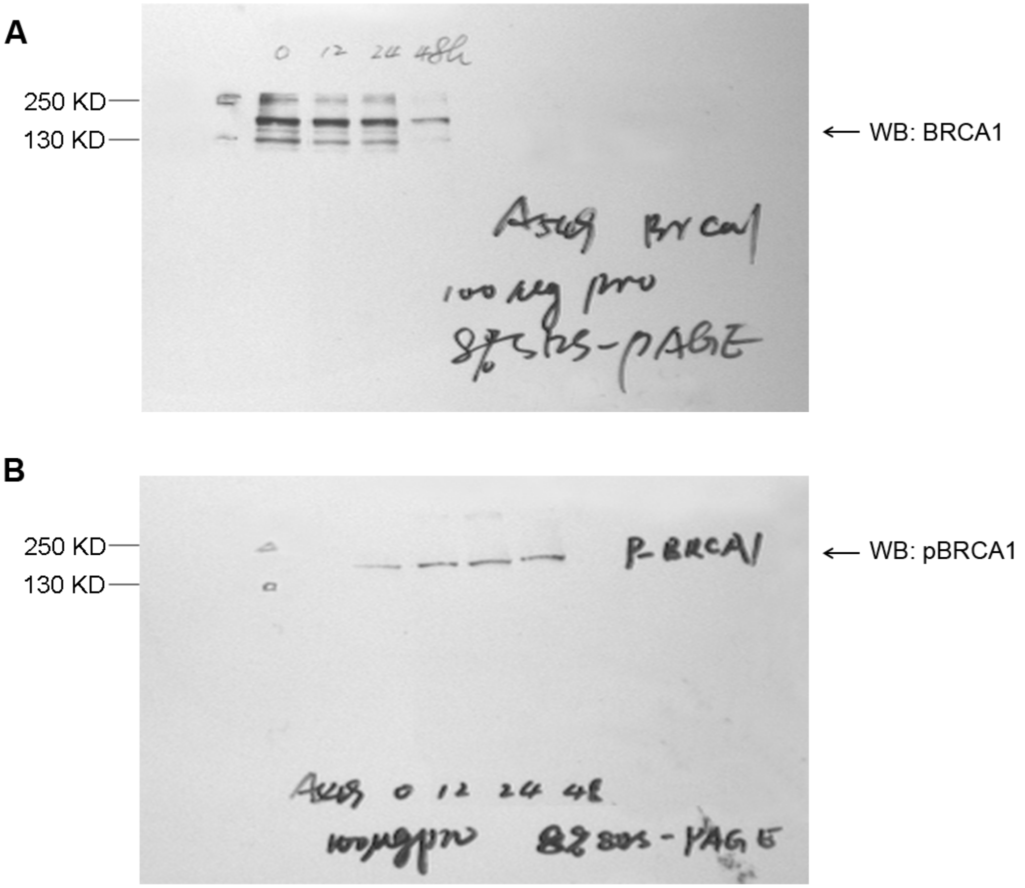

Figure S1. Western blotting for BRCA1/pBRCA1 in A549 cells in Figure 7C. A549 cells were exposed to 2 μM 8-Cl-Ado for the indicated time points (hours). The BRCA1 (**A**) and pBRCA1 (**B**) were analyzed by Western blotting.
